# Maternal Supplementation with Herbal Antioxidants during Pregnancy in Swine

**DOI:** 10.3390/antiox10050658

**Published:** 2021-04-23

**Authors:** Víctor H. Parraguez, Francisco Sales, Oscar A. Peralta, Mónica De los Reyes, Alfonso Campos, Javier González, Wolfgang Peralta, Camila Cabezón, Antonio González-Bulnes

**Affiliations:** 1 Faculty of Veterinary Sciences, University of Chile, 8820808 Santiago, Chile; operalta@uchile.cl (O.A.P.); mdlreyes@uchile.cl (M.D.l.R.); 2 Faculty of Agricultural Sciences, University of Chile, 8820808 Santiago, Chile; 3 INIA-Kampenaike, 6212707 Punta Arenas, Chile; fsales@inia.cl; 4 Agroservice, 7560908 Santiago, Chile; alfonso@agroservice.cl (A.C.); javier@agroservice.cl (J.G.); 5 Agrosuper, 2820000 Rancagua, Chile; wperalta@agrosuper.com (W.P.); ccabezon@agrosuper.com (C.C.); 6 Departamento de Producción y Sanidad Animal, Facultad de Veterinaria, Universidad Cardenal Herrera-CEU, CEU Universities, C/Tirant lo Blanc, 7. Alfara del Patriarca, 46115 Valencia, Spain; antonio.gonzalezbulnes@uchceu.es; 7 Departamento de Toxicología y Farmacología, Facultad de Veterinaria, Universidad Complutense de Madrid, Ciudad Universitaria s/n, 28040 Madrid, Spain

**Keywords:** antioxidants, intrauterine growth restriction, pregnancy, prolificacy, swine

## Abstract

The effects of a combined supplementation with herbal antioxidants during pregnancy on reproductive traits and piglet performance (number of live, dead, and mummified newborns and litter weight at birth and individual body weight at both birth and weaning) were assessed in a total of 1027 sows (504 treated and 523 control females) kept under commercial breeding conditions. The supplementation increased the number of live-born piglets (13.64 ± 0.11 vs. 12.96 ± 0.13 in the controls; *p* = 0.001) and the total litter weight, decreasing the incidence of low-weight piglets without affecting the number of stillbirths and mummified newborns. Such an effect was modulated by the number of parity and the supplementation, with supplementation increasing significantly the number of living newborns in the first, second, sixth, and seventh parities (0.87, 1.10, 1.49, and 2.51 additional piglets, respectively; *p* < 0.05). The evaluation of plasma vitamin concentration and biomarkers of oxidative stress (total antioxidant capacity, TAC, and malondialdehyde concentration, MDA) performed in a subset of farrowing sows and their lighter and heavier piglets showed that plasma levels of both vitamins were significantly higher in the piglets than in their mothers (*p* < 0.05 for vitamin C and *p* < 0.005 for vitamin E), with antioxidant supplementation increasing significantly such concentrations. Concomitantly, there were no differences in maternal TAC but significantly higher values in piglets from supplemented sows (*p* < 0.05). On the other hand, supplementation decreased plasma MDA levels both in the sows and their piglets (*p* < 0.05). Finally, the piglets from supplemented mothers showed a trend for a higher weaning weight (*p* = 0.066) and, specifically, piglets with birth weights above 1 kg showed a 7.4% higher weaning weight (*p* = 0.024). Hence, the results of the present study, with high robustness and translational value by offering data from more than 1000 pregnancies under standard breeding conditions, supports that maternal supplementation with herbal antioxidants during pregnancy significantly improves reproductive efficiency, litter traits, and piglet performance.

## 1. Introduction

Modern swine production is at the head of worldwide meat production, based on large farms implementing efficient genetic selection, health assessment, and nutritional and reproductive strategies. One of the main technical objectives has been focused on selecting lines with higher prolificacy to increase the number of piglets and therefore farm profitability. Such improvement of prolificacy has been mainly achieved by the selection of genotypes with a high ovulatory rate and consequently a high number of developing fetuses.

However, the space into the uterus for the adequate development of the fetuses and their placentae is limited, so large litters are affected by deficiencies of placental development affecting the functionality of the organ and, therefore, the supply of oxygen and nutrients to the fetuses [1]. The consequence is the impairment of fetal growth in a process known as intrauterine growth restriction (IUGR), leading to low birth-weight (LBW) neonates [2,3,4]. Appearance of LBW piglets causes substantial economic losses to farms. Similarly to other species, LBW piglets show higher morbidity and mortality [4,5] but also lower growth potential, lower feed efficiency, lower meat yield, and excess adiposity than their normal-weight littermates [6,7,8,9]. Moreover, these features also reduce the value of farm products by introducing undesirable heterogeneity in growth patterns, carcass conformation, and meat characteristics within the same litter and feedlot [10,11].

The reduced supply of nutrients and oxygen to the IUGR fetuses causes malnutrition and hypoxia which, in turn, increases their oxidative stress [12,13]. Concomitantly, the antioxidant defense system in IUGR fetuses is weakened, which increases oxidative stress and, in turn, aggravates IUGR [14,15,16]. Hence, a possible strategy for counteracting IUGR may be based on the administration of antioxidant agents which ameliorate the antioxidant/oxidative status, improve the placental function, and increase the birth weight and viability of the offspring [17,18]. Specifically, previous studies in sheep with twin pregnancies have shown that maternal oral supplementation with antioxidant vitamins C and E during pregnancy increases these vitamins in the cord blood of near-to-term fetuses, diminishing their oxidative stress and increasing their body weight [19]. Afterwards, the lambs from supplemented ewes have increased plasma antioxidant capacity and higher body weight at birth, improved survival during the lactation period, and showed a trend towards higher body weight at weaning [20].

The administration of vitamin E as an antioxidant agent is a standard practice in modern swine production. However, there is scarce information on the combined supplementation of vitamins C and E during pregnancy. Previous studies under experimental conditions have shown that such a combination improves the antioxidant/oxidative status of the sow [21] and favors the transfer of vitamin E to the piglets and their postnatal growth patterns [22].

These results suggest that supplementation with antioxidant agents may be a promising strategy for diminishing incidence and consequences of IUGR in swine, but translation from basic research to practice may be hampered by environmental and management conditions (i.e., by differences between experimental and commercial farms). Hence, the present study aimed to evaluate the effects of maternal oral supplementation with herbal antioxidants on the antioxidant/oxidative status of sows and piglets and on pregnancy outcomes, litter features, and productivity of the sows under commercial farm conditions. Previous studies on maternal nutritional supplementation in swine (e.g., amino acids) have shown that parity number of the sow influences the response to the supplementation [23], and therefore this variable was also assessed in this study.

## 2. Materials and Methods

### 2.1. Ethics Statement

The study was conducted in a commercial farm in Chile (Agrosuper, Rancagua, Chile) under standard commercial swine breeding conditions. All procedures in this study are consistent with international rules for care and use of animals in research [24], with institutional approval from the University of Chile (reference number 20403–VET–UCH).

### 2.2. Animals and Experimental Design

The study involved 1060 pregnant sows from two different Landrace x Large White-based strains (L-22 and L-42), with number of parities ranging from 1 to 7. All these sows were fed during the entire pregnancy with a standard grain-based food diet adjusted to body weight (a mean of 2.2 kg/day, offered once a day) to fulfill individual daily maintenance requirements based on data from the PIC Nutrient Specifications Manual [25]. This basic diet considers the inclusion of 66 IU of vitamin E per kg of food, while vitamin C is not added. Around half of the sows were selected as the treated group (Group T, *n* = 520), receiving a diet supplemented with herbal-based products with polyphenols mimicking vitamin C and E antioxidant activity (C-Power^®^ and Herbal-E50^®^, respectively; Nuproxa, Switzerland) included as a premix (290 mg of each herbal antioxidant supplement per kg of diet). C-Power^®^ contains *Emblica officinalis* (containing mainly gallic acid) and *Ocimum sanctum* (containing apigenin), while Herbal-E contains *Ocimum sanctum*, *Ocimum basilicum* (containing eugenol) and *Phyllanthus emblica* (containing gallic and tannic acids). The remaining sows acted as controls consuming the same diet without supplementation (Group C, *n* = 540). The treatment was carried out during the entire period in which the females remained in the gestation barns, beginning one day after insemination and ending on day 112 after insemination, when they were transferred to the farrowing room. Housing and handling of the animals were exactly the same in both groups and, for discarding effects during lactation, treated groups returned to the non-supplemented diet two days before the estimated date of farrowing, when they were transferred from the gestation barns to the farrowing rooms.

At birth, the total number of piglets (alive, dead, and mummified), the total weight of the litter, and the individual weight of each piglet were recorded for each sow. Afterwards, individual weight of the piglets was recorded again at weaning (21 days old). Additionally, immediately after farrowing, a representative cluster of 14 litters per group was selected and blood jugular samples were obtained in the sow (10 mL) and two of its piglets (3 mL; selecting one piglet above 1.2 kg of birth weight and one piglet below 1.0 kg of birth weight). After being centrifuged, the plasma was stored in liquid nitrogen to be assayed for concentrations of vitamin C and E, total antioxidant capacity (TAC), and lipid peroxidation (in terms of malondialdehyde concentrations, MDA).

### 2.3. Evaluation of Vitamin Concentrations and Oxidant/Antioxidant Status of Sows and Piglets

Vitamins C and E were measured by high-performance liquid chromatography as previously described [17]. In brief, vitamin C (in the form of ascorbic acid) was measured in plasma samples diluted 10-fold in ultra-pure water, by means of amperometric detection, using a glassy carbon electrode operated at 800 mV and a Ag/AgCl reference electrode. The detection and quantification limits for this assay were 0.1 and 0.2 ug/dL, respectively. Vitamin E (in the form of α-tocopherol) was measured in ethanol/dichloromethane extracted plasma samples by means of spectrofluorimetric detection at 290 and 330 nm wave-length for excitation and emission. The detection and quantification limits for this assay were 0.097 and 0.1 ug/mL, respectively.

Values for total antioxidant capacity (TAC) and malondialdehyde concentrations (MDA) in plasma of sows and piglets were measured with colorimetric kits (Antioxidant Assay Kit and TBARS- TCA, respectively; Cayman Chemical Company, Ann Arbor, MI, USA) using a microplate reader at 405 and 540 nm of absorbance for TAC and MDA, respectively (Perlong DNM-9602; Nanjing Perlove Medical Equipment Co. Ltd., Nanjing, Jiangsu China).

### 2.4. Statistical Analysis

The experimental model included treatment (with and without supplementation), strain (L-22 and L-42), and number of parity (1 to 7), as well as their possible interactions. Comparisons were conducted by analysis of variance, using the general linear model procedure of SAS (GLM; SAS Institute Inc., Cary, NC, USA), after normality testing of the data (Kolmogorov–Smirnov test). There were no significant effects of the strain in any of the parameters evaluated, so such a factor was excluded from the posterior analyses. Data from pregnancies with litter size exceeding 2 SD above or under the mean value were excluded from these analyses (17 pregnancies, 3.1% of total pregnancies, in Group C and 16, 3.0% of total pregnancies, in Group T). Hence, 523 control and 504 treated pregnancies were studied (Table 1). Finally, the degree of association between plasma concentrations of vitamins C and E and oxidative stress biomarkers were evaluated using the Pearson correlation analysis. All the results were expressed as mean ± S.E.M. Statistical significance was accepted from *p* < 0.05 and a statistical trend was considered when 0.05 < *p* < 0.1.

## 3. Results

### 3.1. Effects of Supplementation on Plasma Vitamin Concentrations in Sows and Piglets

**Figure 1:**
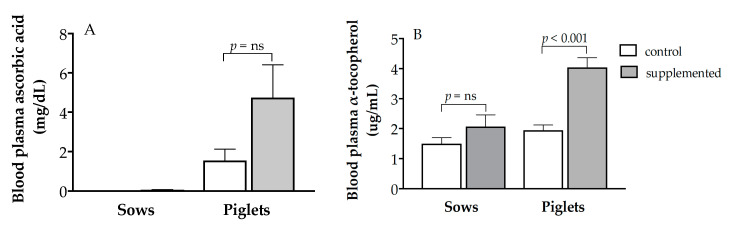
Mean values (± S.E.M.) of plasma concentrations of vitamin C (panel **A**) vitamin E (panel **B**) at day of farrowing in sows and newborn piglets treated or not with herbal antioxidants. ns = not significant.

Assessment of plasma vitamin concentrations showed non-detectable concentrations of vitamin C in the control sows and almost non-detectable concentrations in the treated females (Figure 1A; Groups C and T, respectively). Meanwhile, plasma concentrations of vitamin E showed a non-significantly numerically higher value in Group T (2.08 ± 0.39 vs. 1.05 ± 0.19 µg/mL in Group C; Figure 1B).

Vitamin concentration in the blood plasma of heavier and lighter piglets showed no differences between piglets < 1 and > 1.2 kg of body weight for both vitamin C and E. Hence, independently of birth weight, piglets in Group T showed numerically higher values of vitamin C (4.74 ± 1.66 vs. 1.54 ± 0.58 mg/dL in Group C; Figure 1A) and significantly higher values of vitamin E (4.05 ± 0.32 vs. 1.95 ± 0.18 µg/mL in Group C, *p* < 0.001; Figure 1B). The plasma levels of both vitamins were significantly higher in the piglets than in their mothers (*p* < 0.05 for vitamin C and *p* < 0.005 for vitamin E).

### 3.2. Effects of Supplementation on the Antioxidant Capacity and Oxidative Stress of Sows and Piglets

**Figure 2:**
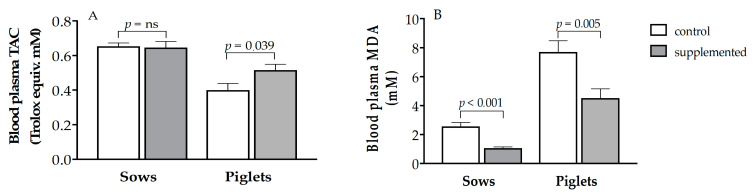
Mean values (± S.E.M.) of plasma total antioxidant capacity (TAC; panel **A**) and malondialdehyde (MDA; panel **B**) at day of farrowing in sows and newborn piglets treated or not with herbal antioxidants. ns = not significant.

Evaluation of TAC and MDA in blood plasma of piglets showed no differences between light and heavy individuals, so the results of oxidative stress in piglets are presented without differentiating between body-weight groups. The herbal supplementation showed no effects on TAC of sows but a significant increase in their piglets (*p* < 0.05; Figure 2, panel A). Conversely, the treatment decreased plasma concentrations of MDA in both sows and piglets (*p* < 0.005 and *p* < 0.01, respectively; Figure 2, panel B). The comparison of both parameters between sows and piglets evidenced a higher pro-oxidative status in the piglets than in their mothers (*p* < 0.001), which was partially alleviated in treated piglets (*p* < 0.05). In this sense, there were significant relationships between concentrations of both vitamins and TAC in the plasma of newborn piglets (*r* = 0.538, *p* < 0.05, for vitamin C and *r* = 0.786, *p* < 0.001, for vitamin E). Higher plasma concentrations of vitamin E were also associated to lower MDA plasma concentrations (*r* = −0.751, *p* < 0.001).

### 3.3. Effects of Maternal Supplementation on Litter Features at Farrowing

**Figure 3:**
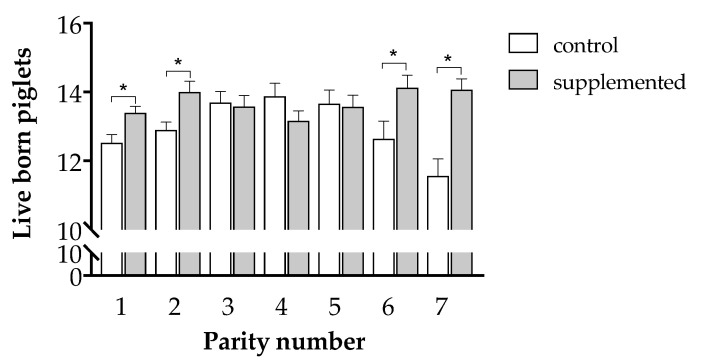
Mean values (± S.E.M.) of living piglets according to parity in sows treated or not with herbal antioxidants. Asterisks denote significant differences between groups (*p* < 0.05).

The characteristics of the litters were positively influenced by the maternal supplementation. Accordingly, the number of living newborns was increased in 0.67 piglets (12.96 ± 0.13 in Group C and 13.64 ± 0.11 in Group T; *p* < 0.001). Such effect was modulated by the number of parity. In control sows, the third, fourth, and fifth parities had the highest litter size, whilst first and seventh parities had the lowest litter size (*p* < 0.05). The supplementation increased significantly the number of living newborns in the first, second, sixth, and seventh parities (0.87, 1.10, 1.49, and 2.51 additional piglets, respectively; *p* < 0.05; Figure 3). Conversely, there were no significant effects in the number of stillborn (0.22 ± 0.02 vs. 0.24 ± 0.03 for Groups T and C, respectively; *p = ns*) or mummified piglets (0.34 ± 0.04 vs. 0.33 ± 0.04 for Groups T and C, respectively; *p = ns*).

**Figure 4:**
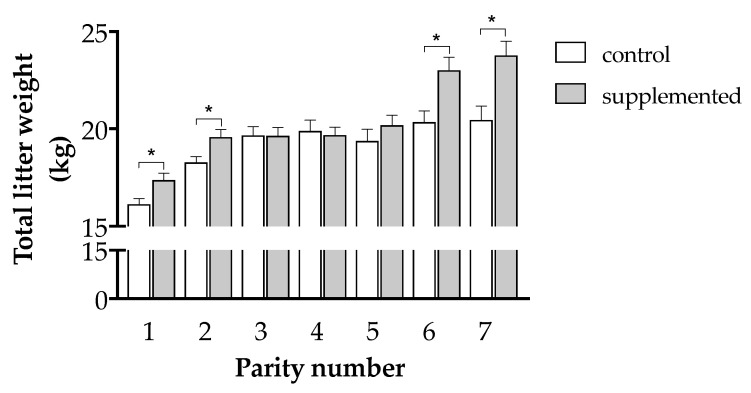
Mean (± S.E.M.) litter weight according to parity in sows treated or not with herbal antioxidants. Asterisks denote significant differences between groups (*p* < 0.05).

Maternal supplementation also improved the total weight of the litter and diminished the incidence of low birth-weight piglets. The differences between groups in the mean individual birth weight of the piglets showed no significant differences, but the total litter weight increased around 1.25 kg in treated sows (19.7 ± 0.2 vs. 18.5 ± 0.2 kg in Groups T and C, respectively; *p* < 0.001). Again, supplementation increased significantly the total litter weight in the first, second, sixth, and seventh parities (1.24, 1.30, 2.65, and 3.33 additional kg, respectively; *p* < 0.05; Figure 4), but not in the third to fifth parities.

**Figure 5:**
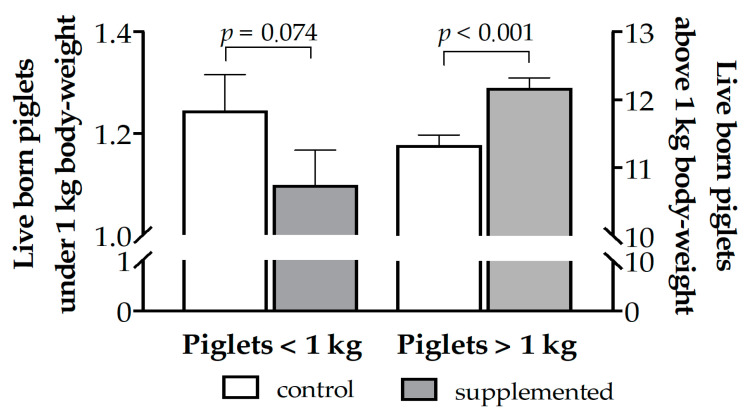
Mean number (± S.E.M.) of piglets under and above 1.0 kg of birth weight in sows treated or not with herbal antioxidants.

The treated group showed a trend for a decrease in the total number of piglets with a birth weight under 1 kg (*p* = 0.07), with its incidence being around 9% lower than in the controls due to a significantly higher number of piglets with a birth weight above 1 kg (around 0.84 additional piglet per litter; *p* < 0.001; Figure 5).

### 3.4. Effects of Maternal Supplementation on Postnatal Patterns of Growth and Development of Offspring

**Figure 6:**
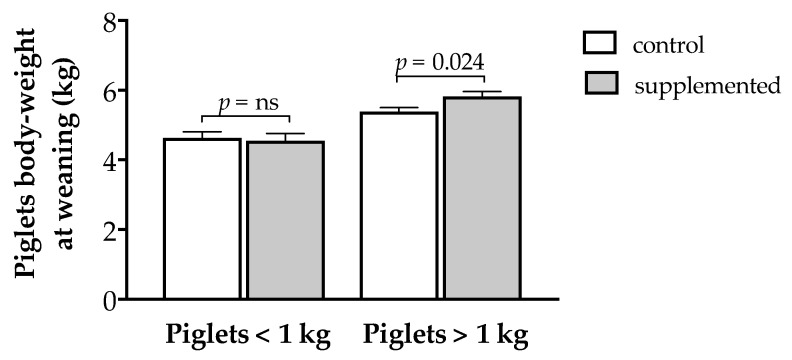
Mean weaning weight (± S.E.M.) of piglets with birth weights under and above 1 kg in sows treated or not with herbal antioxidants. ns = not significant.

The piglets from sows with herbal supplementation during pregnancy showed a trend for a higher body weight at weaning (5.41 ± 0.13 vs. 5.09 ± 0.11 in Groups T and C, respectively; *p* = 0.066). Such lack of significant differences was related to a different evolution in piglets with birth weights under or above 1 kg (Figure 6). There were no significant differences in the case of piglets with birth weights under 1 kg, but piglets from Group T with birth weights above 1 kg showed a 7.4% higher weaning weight (5.81 ± 0.15 vs. 5.39 ± 0.12 kg in Groups T and C, respectively; *p* = 0.024).

## 4. Discussion

There are a limited number of articles reporting the effects of antioxidants on reproductive and productive traits in pigs, and most of them are conducted with a small number of animals at experimental flocks. To our knowledge, this is the first trial using a large number of pregnant sows to study the effects of supplementation of herbal antioxidants on the pregnancy outcome and the growth of lactating piglets. The main results of the present study show that supplementation with herbal antioxidants during pregnancy leads to a significant increase in the number of live born piglets and a consequent increase in litter weight; mainly explained by the increase in the number of live born piglets with body weight greater than 1 kg. Such group of piglets also achieved a significant increase in body weight at weaning.

The results of the present study showed, firstly, that maternal supplementation with herbal antioxidants during almost the entire pregnancy resulted in a differential expression of the oxidative status biomarkers in blood plasma, both in sows and piglets.

The nutritional requirements of vitamin C in pigs have not really been defined, since it is assumed that they synthesize it in adequate quantities, but it is accepted that, under high-demanding conditions, supplementation may have beneficial effects [25]. However, in our study, plasma vitamin C was almost undetectable in the sows and, although higher concentrations were detected in the treated group, there were no significant effects of the supplementation. Similar results have been previously reported [26] in non-supplemented and counterpart supplemented (1 g of vitamin C per day) pregnant sows. In such a study, significant increases in plasma vitamin C concentrations were only reported when the dose was 10-fold higher (10 g per day) and, despite the low maternal plasma concentrations of vitamin C, the concentrations of the vitamin in the plasma of the newborns were substantially higher, about 20- and 30-fold higher than in their mothers in the control and treated groups, respectively. These differences were consistent with other data previously reported [27,28]. In brief, vitamin C concentrations in the fetus increases during late gestation, but serum and liver ascorbic acid concentration declines in the sow, presumptively by an increased transfer of vitamin C to the fetus and to the mammary gland [28]. The low plasma concentrations of vitamin C in the sow and the high concentration of the vitamin in the offspring have been explained by a large maternal–fetal transfer through the placenta, which is retained by the fetus [27,29]. Placental vitamin C transport maintains higher vitamin C concentration on the fetal side of the placenta than on the maternal side and such transport is mediated at the placenta by a specific transporter termed Sodium-dependent Vitamin C Transporter 2 (SVCT2) [30]. Afterwards, there is also a large amount of vitamin C transferred from maternal plasma to colostrum in the case of the newborn. In fact, vitamin C in sow colostrum reaches more than 30 mg/dL, which is about 50-fold higher than in maternal plasma [26].

Plasma concentrations of vitamin E found in our study were also consistent with a previous experiment using similar daily maternal vitamin supplementation but in a shorter treatment that started at day 90 of gestation [31]; plasma vitamin E concentration in supplemented sows were higher than in the controls, but differences did not reach statistical significance. Statistical differences were only found when supplementing with very high doses during the entire pregnancy [32]. The assessment of plasma vitamin E concentrations showed, similarly to vitamin C, that piglets had higher values (about two-fold) than their mothers in both supplemented and control groups; which is again in agreement with previous studies [33,34], although others did not report differences [30]. Furthermore, there was a significant increase of vitamin E in piglets from supplemented mothers, which is also consistent with previous reports [33,35], in spite of the low transfer capacity of the swine placenta for vitamin E [22,36].

The treatment with herbal antioxidants had an attenuating effect on maternal oxidative stress, as indicated by the significant reduction in plasma MDA concentrations, in a similar way to treatments with vitamins C and E plus either β-carotene [21] or selenium [37]. On the other hand, there are no previous reports, to the best of our knowledge, on the effects of supplementing pregnant females with a combination of herbal antioxidants on the antioxidant status of their piglets. Our data show changes of remarkable magnitude, either by the assessment of TAC or MDA. Such improvement in oxidative status was related to the high concentration of antioxidant vitamins reached by the piglets. These results suggest that supplementation with herbal antioxidants may be a very promising strategy for improving health status and development of newborns, having in mind the high level of oxidative stress occurring in sows during late gestation [38,39] and in piglets around farrowing [40].

Importantly, the assessment of productive traits showed that the supplementation of sows during pregnancy increases the number of live-born piglets and the total litter weight, decreasing the incidence of low-weight piglets, without affecting the number of stillbirths and mummified newborns.

Previous studies have shown contradictory results, with positive [37] or no effects [31], which may be influenced by the duration of the treatment and therefore the pregnancy stages influenced by the supplementation. Maternal oxidative stress has been previously associated with decreased reproductive success in sows, in terms of litter size and piglet weight [41]. The adverse effects of oxidative stress on fertility and offspring development can be located at different levels, from ovarian function to implantation and/or embryo/fetal development and there is little information regarding specific reproductive processes or functions disturbed by oxidative stress in pigs. In women, significant relationships between high oxidative stress and alterations in follicular and embryonic development have been described [42,43]. Sows can ovulate between 15 and 30 oocytes in each estrous cycle, in a very short period of no more than 3 h [44]. Therefore, the probability of oocyte fertilization is high, ruling out possible asynchrony between intercourse or insemination and the rate of fertilized oocytes. Issues related to a better embryo survival and developmental rate both at pre-implantational stages [45,46] and/or after implantation [46,47,48] are more definitive for reproductive success. In this sense, significant antiapoptotic and embryotrophic effects have been found after addition of vitamins C or E to culture media during porcine in vitro embryo production [45]; however, these positive effects were not found when both vitamins were used simultaneously, which may indicate an excessive total dose for a balanced antioxidant status.

Other in vivo studies have shown that the use of only vitamin E but combined with selenium induced an increase in litter size and birth weight of piglets, while supplementation only with vitamin E had no effects [33]. The absence of effect of vitamin E alone, however, may be due to the fact that the dose used (50 mg/kg of feed), although it is within the range suggested by NCR [25] to maximize litter size and immunocompetence of piglets, may be too low to achieve an antioxidant effect when oxidative stress is high. In contrast, supplementation of sows of first and second parity with vitamin E at doses of 40 and 70 IU/kg of feed induced dose-dependent increases in litter size with a higher number of live-born piglets and lower number of stillbirths [34]. These results evidenced the importance of the parity of the sow on the response to antioxidants.

In our present study, supporting such importance of parity, the positive effects of supplementation were mostly expressed in parities 1, 2, 6, and 7. Nutrient requirements of pregnant sows, and dietary antioxidant requirements, are affected by parity of sows [49,50]. First-parity gilts and second-parity sows are characterized by a compromised homeostasis [51], since first-parity gilts need to fulfill requirements for both fetal growth during pregnancy and their own growth to reach a mature body size [52,53]. Second-parity sows often have lower reproductive performance than mature sows, and even than first-parity gilts [54] due to the metabolic challenge of the first lactation over the following reproductive cycles [55]. Older sows have compromised metabolic and reproductive features and mean parity for culling sows is five [56]. The consequences are similar when considering oxidative homeostasis, since catabolic condition reduces antioxidant defenses and therefore increases oxidative stress in the mother sow, mainly during late gestation and lactation [38]. These changes are modulated by aging, nutritional status stress, and disease [50]. The higher oxidative conditions in replacement gilts than in multiparous sows, due to lower mRNA expression and enzymatic activity of major antioxidants, has been directly shown [57] but, moreover, the position in both the low and high social-rank of younger and older sows, those with lower reproductive features, has been found related to higher oxidative stress during late gestation and lactation than in sows in the middle social rank [58]. Hence, supplementation with antioxidants should be, and it was found in our study, more successful in compromised gilts and sows.

Maternal supplementation with antioxidants in the present study improved the growth patterns of the piglets during lactation, reaching higher weaning weight. This effect was fundamentally influenced by piglets that were born weighing more than 1 kg, confirming the importance of birth weight on the postnatal growth trajectory of the piglets [59]. Increased weaning weights have been also described in piglets born to sows supplemented with vitamin E alone [33,34] and vitamin E plus selenium during gestation and lactation [33]. Conversely, maternal supplementation with vitamin E alone or with combined vitamins C and E from 90 days of gestation and during lactation did not show significant effects on the weight of the piglets at weaning [31]. Similar results were obtained when sows were treated with vitamin E plus selenium, vitamin C alone, or the combination of both during the last 15 days of pregnancy [37].

## 5. Conclusions

The results of this study indicate that supplementation of sows throughout gestation with herbal antioxidants significantly improves reproductive efficiency, litter characteristics, and piglet performance during the birth-weaning period. It should be noted that this study was carried out with more than 1000 pregnancies, under standard production conditions, so the results have high reliability and applicability to improve the productivity of pig systems at different scales.

## Figures and Tables

**Table 1:** Summary of the distribution, by treatment and number of parity, of the pregnancies assessed in the present study.

Group	Number of Parity						
	1	2	3	4	5	6	7
**Control**	148	111	75	50	54	47	38
**Treated**	136	86	73	70	51	47	41
**Total**	284	197	148	120	105	94	79

## Data Availability

The data presented in this study are available on request from the corresponding author. The data are not publicly available due to privacy.
